# Effect of Flash Lamp and Furnace Annealing on the Electrical and Optical Properties of Ti–Al-Codoped ZnO Films Deposited by DC Magnetron Sputtering

**DOI:** 10.1021/acsomega.5c11669

**Published:** 2026-05-26

**Authors:** Mingyu Kim, Guoxiu Zhang, Yoon-Young Huh, Chang-Hyeon Jo, Fabian Ganss, Mohsin Saleem, Shengqiang Zhou, Jung-Hyuk Koh, Slawomir Prucnal

**Affiliations:** † Department of Intelligent Energy and Industry, 26729 Chung-Ang University, Heukseok-ro, Dongjak-gu, Seoul 06974, Republic of Korea; ‡ School of Electrical and Electronic Engineering, Chung-Ang University, Seoul 06974, Republic of Korea; § Helmholtz-Zentrum Dresden-Rossendorf, Institute of Ion Beam Physics and Materials Research, Bautzner Landstraße 400, 01328 Dresden, Germany; ∥ Dresden University of Technology, 01062 Dresden, Germany; ⊥ School of Chemical and Materials Engineering (SCME), National University of Sciences & Technology (NUST), H-12, Islamabad 44000, Pakistan

## Abstract

Aluminum-doped zinc oxide (AZO) is one of the most commonly used transparent conductive oxides because of its low resistivity and high transmittance. AZO is an excellent candidate for transparent electronic devices, photovoltaics, and thin-film technologies. However, the resistivity and ultraviolet–visible (UV–vis) transmission of AZO are still significantly inferior to those of indium tin oxide (ITO). Two things are needed for the practical application of AZO: low resistance and low production costs. One method of reducing the resistance of the layer is codoping AZO with titanium, while lower production costs can be achieved by using more efficient technological processes. In this work, we investigated the influence of titanium codoping and millisecond-range flash lamp annealing (FLA) on the structural and optoelectronic properties of Ti–Al-doped ZnO (TAZO) thin films fabricated by magnetron sputter deposition. Ti was codeposited into the AZO films by placing Ti pellets on top of the AZO target during magnetron sputtering. Postdeposition annealing was performed using either conventional furnace annealing (FA) for 30 min or FLA for 23 ms. Our results show that thermal treatment by millisecond-range FLA improves the electrical conductivity of TAZO by a factor of approximately 100 while maintaining an average transmittance of over 80%. At the same time, FLA requires much less energy than conventional heating, which makes it more economically efficient. By directly comparing FA and optimized FLA, we demonstrate that FLA-treated samples achieve similar or superior crystallinity and a 1 order of magnitude lower sheet resistance while preserving comparable optical transmittance. These results demonstrate that the combination of FLA and Ti codoping significantly enhances the performance of TAZO thin films, making them promising candidates for transparent electrode applications in optoelectronic devices.

## Graphical abstract

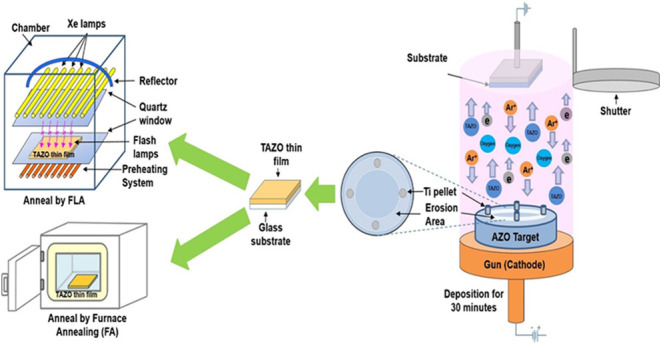

## 1 Introduction

Transparent conductive oxides (TCOs) have garnered significant interest owing to their high electrical conductivity and high transmission in the ultraviolet–visible (UV–vis) spectral range, which makes them suitable for widespread applications in optoelectronic devices such as solar cells, flat or flexible panel displays (e.g., LCDs and touchscreens), light-emitting diodes (LEDs), and gas sensors.^1–4^ Currently, one of the most popular TCO materials is indium tin oxide (ITO) due to its high electrical conductivity and superior optical properties in the UV–vis spectral range of 400–800 nm. Unfortunately, indium belongs to the family of critical raw materials, making it expensive. Moreover, indium mining technology is very harmful to the environment.^5^ Therefore, there is a strong need to replace ITO with other TCOs that have similar optoelectronic properties but are cheaper and more environmentally friendly. Among these, zinc oxide (ZnO) has attracted interest owing to its high conductivity, nontoxicity, and zinc’s high abundance. It also offers ease of synthesis, along with high thermal and chemical stability. ZnO is a direct band gap semiconductor with *E*_g_ ≈ 3.3 eV and a large excitonic binding energy (60 meV) that makes it attractive for LEDs.^6,7^ Many different deposition techniques offer good substrate adhesion even at room temperature, which makes ZnO attractive for integration with different functional devices, such as an electron-transporting layer for perovskite light-emitting diodes^8^ and solar cells.^9,10^ Nevertheless, the lower conductivity of ZnO compared to that of ITO is a major problem in the way of the full replacement of ITO by ZnO. Therefore, further enhancements in conductivity and transparency are required for their practical applications. The electrical conductivity of ZnO can be increased by defect engineering, such as oxygen vacancies (V_O_) and Zn interstitials (I_Zn_), or by controlled doping with group-III elements (Al^3+^, Ga^3+^, In^3+^, B^3+^), transition metals (Co^2+^, Ni^2+^, Mn^2+^, Cu^2+^, Ti^4+^), or tin and fluorine.^11–13^ Among these, Al is considered an abundant and nontoxic dopant for ZnO. ZnO doped with Al has a resistivity on the order of 10^–4^ Ω·cm and has already found application in solar cells and antireflection coatings for low-emissivity glass windows.^14^ Despite enormous research efforts, the optoelectronic properties of AZO are still inferior to those of ITO, which means that new methods for improving AZO are constantly being sought. Besides Al, titanium can be cosputtered and used as a donor in ZnO. The tetravalent cation Ti^4+^ with an ionic radius of 0.68 Å has a smaller radius than the divalent Zn^2+^ (0.74 Å), and when it replaces Zn, it acts as a donor, resulting in higher conductivity.^15^ Moreover, due to its smaller ionic radius, the incorporation of Ti into ZnO can be used for strain engineering. However, an excess concentration above a certain critical level may deteriorate the electrical conductivity since Ti acts as a scattering site.^16–18^ For the deposition of uniform, strongly adhesive thin films, DC sputtering is the most promising deposition technique.^19^ Besides doping, defect engineering plays a crucial role in enhancing the conductivity and transmittance of ZnO. Zinc interstitials (Zn_i_) generally behave as shallow donors that enhance electron concentration, while zinc vacancies (V_Zn_) and oxygen interstitials (O_i_) act as deep acceptors, thereby compensating for n-type doping, and oxygen vacancies (V_O_) serve as carrier traps or deep double donors, depending on their ionization state.^13,34,35^ To provide a well-defined starting point for studying the effect of postdeposition annealing, we deliberately designed our DC sputtering process to operate under oxygen-rich conditions (Ar/O_2_ = 25/5 sccm) at a relatively high working pressure of 4.0 × 10^–2^ Torr. Such conditions enhance the formation of oxygen interstitials and suppress oxygen vacancies, leading to a relatively low carrier concentration and high resistivity in the as-deposited film. This regime is therefore suboptimal for achieving record-low resistivities, but is very sensitive to defect rearrangement and thus ideally suited for comparing furnace annealing (FA) and flash lamp annealing (FLA).^31,32^ In this study, we focus on Ti–Al-codoped zinc oxide (TAZO) thin films deposited by reactive DC magnetron sputtering under oxygen-rich and relatively high-pressure conditions. Rather than optimizing the absolute resistivity, we aimed to use a fixed oxygen-rich growth regime that is representative of large-area industrial processes to systematically compare conventional furnace annealing (FA) and millisecond-range flash lamp annealing (FLA) as postdeposition treatments. Titanium is introduced via Ti pellets mounted on the erosion track of an AZO sputter target, enabling precise control of the Ti content from P0 to P4 by adjusting the number of pellets from zero to four. After deposition, the samples were annealed either by FLA for 23 ms at different energy densities or by FA at 500 °C for 30 min. For clarity, the Ti-free sample is referred to as AZO (TAZO-P0), while the Ti–Al-codoped samples are denoted as TAZO-P1 to TAZO-P4. We use (T)­AZO when referring to the entire series collectively.

The applicable temperature during the FA process is limited by the plastic deformation of the glass substrate at higher temperatures. FLA is a nonequilibrium annealing technique that delivers short-duration, high-intensity light pulses, enabling rapid crystallization and defect reduction without excessive thermal diffusion of dopants.^20^ Therefore, the peak temperature at the sample surface can be much higher than the substrate’s threshold temperature. According to the experimental results, both the optoelectronic and structural properties of the flashed films are significantly improved compared to those of the FA-treated films. We show that a single 23 ms FLA pulse improves the sheet resistance of TAZO by approximately 2 orders of magnitude compared with the as-deposited films and by about 1 order of magnitude compared with FA, while maintaining an average optical transmittance above 80%. By correlating X-ray diffraction (XRD), Field emission scanning electron microscopy (FESEM), UV–Vis transmission, PL, and Hall measurements, we discuss how Ti codoping and the choice of annealing route jointly govern the crystallinity, defect structure, and optoelectronic properties of TAZO films. Room-temperature photoluminescence (PL) clearly shows that FLA in a nitrogen ambient suppresses defect formation, thereby enhancing carrier mobility. These findings contribute to the development of highly efficient TCO materials for next-generation electronic and photovoltaic applications.

## 2 Results

### 2.1 Structural Characterization

The crystallinity of the (T)­AZO films was investigated using X-ray diffraction (XRD), and the resulting patterns are shown in Figure 1. We prepared five samples with Ti concentrations varying from 0 to 0.43 atom % by adjusting the number of Ti pellets mounted on the AZO sputtering target from zero to four. All diffraction peaks of the (T)­AZO films can be indexed to the hexagonal wurtzite ZnO structure (JCPDS card No. 36-1451), and no secondary phases related to Ti or Al oxides are detected. The (100), (002), (101), and (110) reflections are observed at around 31.7°, 34.4°, 36.3–36.7°, and 56–57°, respectively. In the as-deposited pure AZO film, the (100) and (002) reflections appear as the best-defined peaks, whereas the (101) and (110) reflections are comparatively weaker. Here, “best-defined” refers to their relative intensity and visibility within our θ–2θ scans; although the absolute peak intensities are moderate due to the ∼1.2 μm film thickness, the intensity ratios between the (100), (002), and the weaker (101), (110) reflections are sufficient to evaluate the preferred orientation and crystallinity, consistent with previous reports on the AZO and ZnO thin films.^22,23^ After codoping with Ti, the X-ray diffraction pattern is dominated by the (002) reflection, and the (110) reflection becomes hardly visible, indicating that Ti incorporation promotes *c*-axis-oriented growth. After both FLA and FA, all samples except TAZO-P3 show a further increase of the (002) reflection, indicating an improvement in the layer crystallinity. After FLA at 62 and 70.6 J cm^–2^, the enhancement factor I_002_/I_002,as_ reaches approximately 1.50 and 3.22, respectively, for TAZO-P0, is between about 0.95 and 1.6 for the intermediate Ti concentrations (P1–P3), and attains ∼1.78 and ∼1.67 for the most heavily doped sample TAZO-P4.

**Figure 1:**
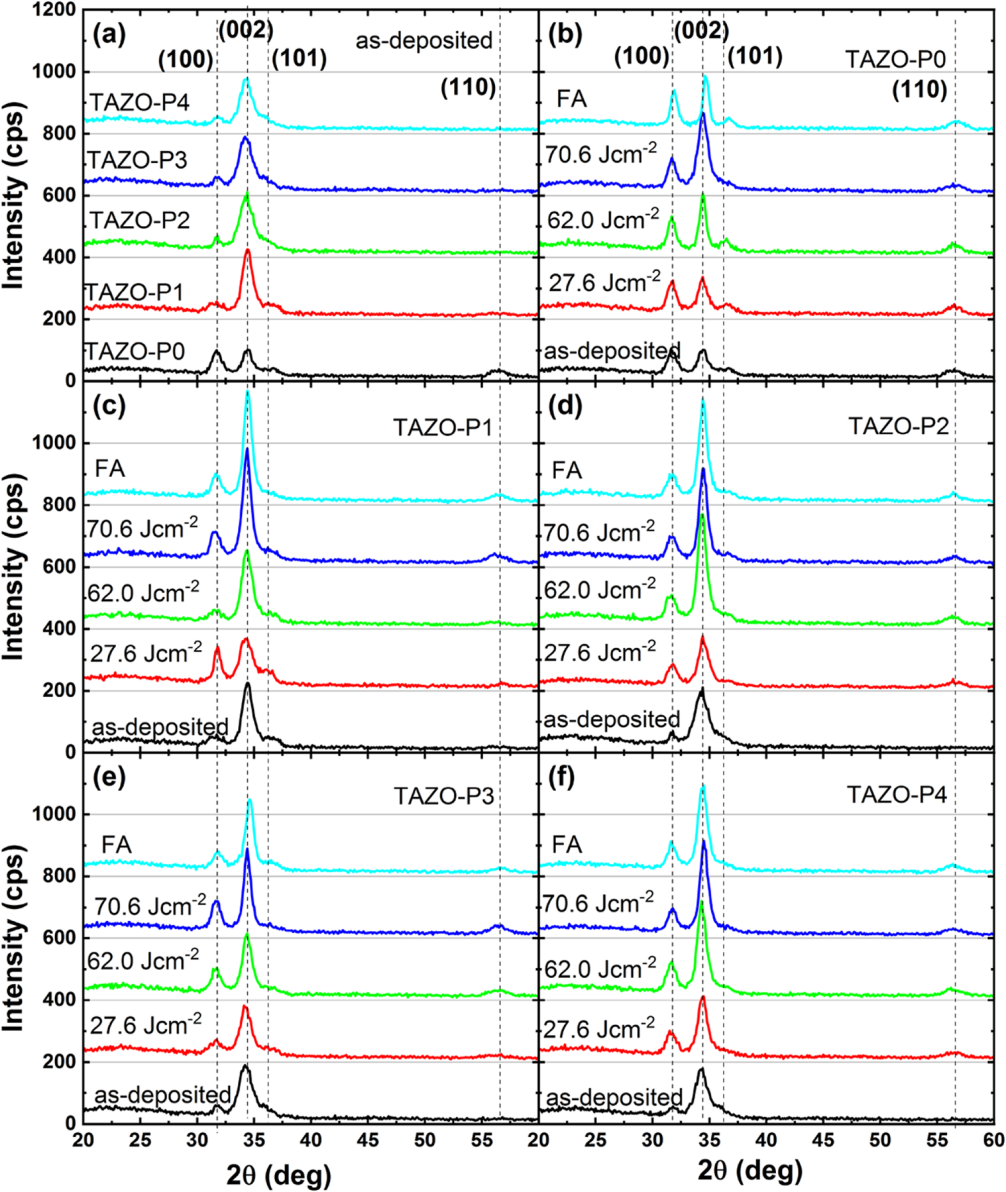
XRD patterns of as-deposited (T)­AZO films (a) and those annealed either by FLA for 23 ms at different energy densities or by furnace annealing for 30 min at 500 °C (b–f). Annealing was performed in a continuous flow of N_2_ gas, and figures (b–f) show the films with increasing Ti concentration. The main peaks are indexed to the (100), (002), (101), and (110) planes of hexagonal wurtzite ZnO (JCPDS card No. 36-1451).

Furnace annealing at 500 °C leads to more moderate changes, with I_002_/I_002,as_ values of 1.25, 1.48, 1.16, and 1.69 for P0, P1, P2, and P4, respectively, while P3 remains almost unchanged (0.93). These trends are consistent with the reduced FWHM (see Figure S1) and enlarged crystallite size presented in Figure 2 and demonstrate that, at the optimized energy density, FLA provides an enhancement of the (002) peak area that is comparable to or, for some compositions, even higher than that obtained by conventional furnace annealing. The integrated intensities of the X-ray diffraction peak intensities and the complete set of I_002_/I_002,as_ values for all compositions and annealing conditions, are summarized in Supporting Tables S1 and S2.

**Figure 2:**
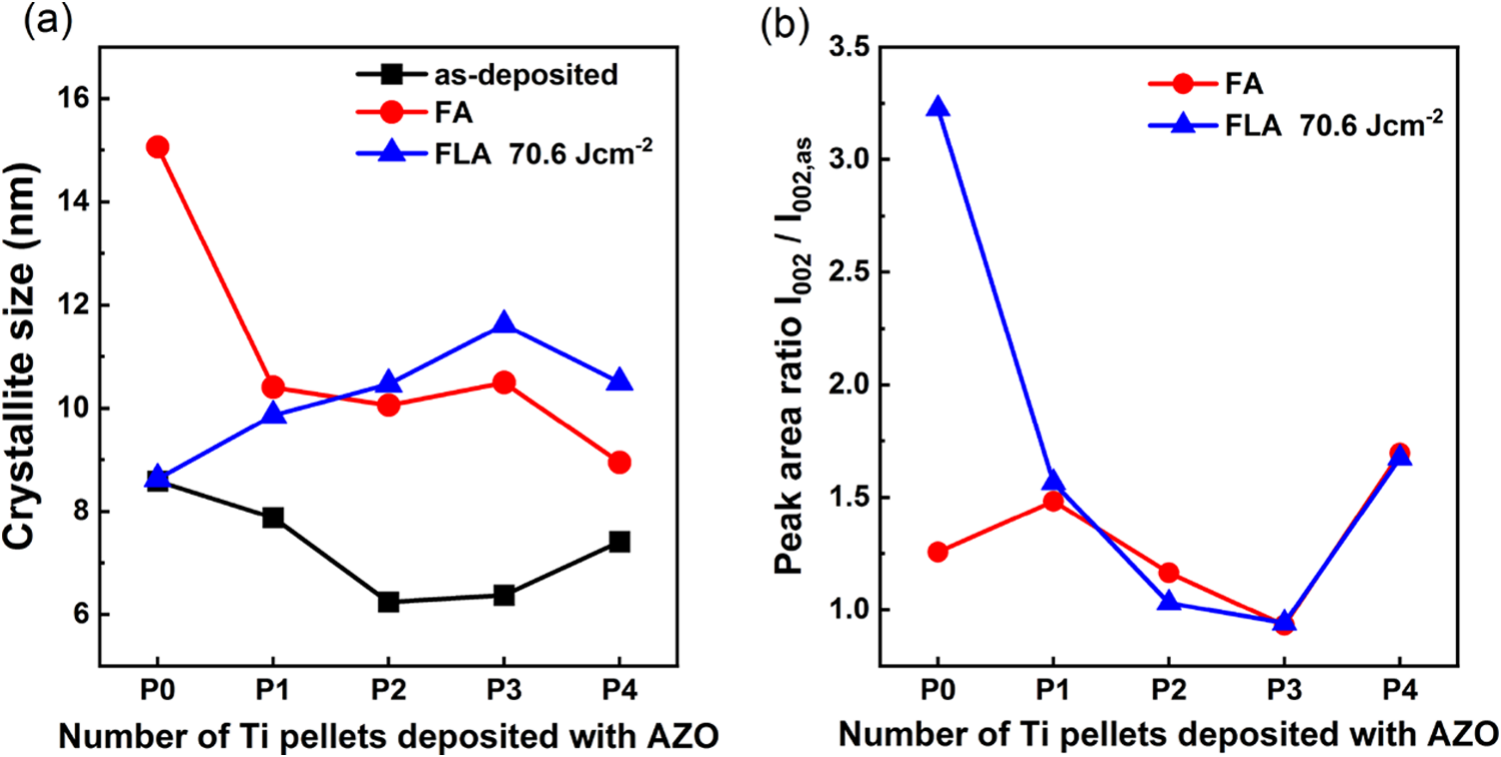
Structural parameters extracted from the ZnO (002) reflection of (T)­AZO films as a function of Ti content (P0–P4): (a) average crystallite size D obtained from the Scherrer equation, and (b) peak area ratio of the ZnO (002) reflection, I_002_/I_002,as_, where I_002,as_ is the integrated (002) intensity (peak area) of the corresponding as-deposited film. Furnace annealing (FA) was performed at 500 °C for 30 min, and flash lamp annealing (FLA) was performed at an energy density of 70.6 J cm^–2^ for 23 ms.

Overall, the dominance of the (002) reflection after Ti codoping and postannealing over the comparatively weak (100), (101), and (110) peaks indicates a significantly preferred orientation of the *c*-axis in the growth direction. This behavior is consistent with the nanocolumnar, *c*-axis-oriented growth commonly reported for the AZO and ZnO thin films.^22,23^ After furnace annealing for 30 min at 500 °C, the X-ray diffraction pattern for all samples is similar to the data obtained from samples annealed by FLA at 70.6 J cm^–2^. Since direct measurements of the film temperature during the FLA process are not possible, we can only try to estimate the maximum achieved temperature by comparing, e.g., X-ray diffraction data. Hence, the peak temperature in the oxide during annealing with 70.6 J cm^–2^ is assumed to be higher than 500 °C.

To quantify the average crystallite size of the (T)­AZO films in the growth direction, we analyzed the full width at half-maximum (FWHM) of the ZnO (002) diffraction peaks presented in Figure S1, and estimated the average crystallite size using the Scherrer formula

1 $$D=\frac{0.9\lambda }{\beta \cos\theta }$$

where *D* is the estimated average crystallite size, λ is the X-ray wavelength (Cu-Kα, 1.54 Å), β is the FWHM of the analyzed peak, and θ is the Bragg angle. Furthermore, we evaluated the integrated intensity ratio of the ZnO (002) reflection with respect to the as-deposited film to track changes in the fraction of the preferentially oriented, crystalline material. Figure 2a,b summarizes the derived crystallite size and the (002) peak area ratio I_002_/I_002,as_, as a function of Ti content for the as-deposited films and for the samples annealed by FA and by FLA at 70.6 J cm^–2^.

For the as-deposited films, the FWHM of the (002) peak is about 1.0° for AZO (P0) and slightly larger (∼1.1°) for TAZO-P1, increasing to a maximum of ∼1.35–1.40° for the intermediate Ti contents P2–P3, and then decreasing again to ∼1.2° for P4. The corresponding crystallite size decreases from about 8–9 nm (P0–P1) to ∼ 6–6.5 nm for P2–P3 and partially recovers to ∼7–7.5 nm for P4. Thus, Ti incorporation beyond a small amount (P1) generally broadens the (002) reflection and suppresses grain growth in the as-deposited state, with only modest recovery at the highest Ti content.

After FLA at 70.6 J cm^–2^, all samples show a marked improvement in long-range order: the FWHM values converge to ∼0.75–1.01°, and the average crystallite size increases to ∼ 8.6–11.6 nm, with only a weak dependence on the Ti content. Furnace annealing at 500 °C also enhances the long-range order, yielding intermediate FWHM (∼0.58–0.97°) and crystallite sizes (∼9–15.1 nm) that are comparable or slightly improved than those obtained after FLA and clearly improved compared with the as-deposited state, demonstrating that millisecond FLA can approach the structural modification obtained by 30 min of furnace annealing. Figure 2b displays the peak area ratio of the (002) reflection, I_002_/I_002,as_, where I_002,as_ is the peak area of the corresponding as-deposited film. For all Ti contents, both FA and especially FLA increase the (002) peak area. At the highest FLA energy density, the enhancement reaches about 3-fold for P0, while the Ti-containing films (P1–P4) show a more moderate response (0.94–1.67), including a nearly unchanged value for P3, indicating an increase in the crystalline volume featuring a *c*-axis fiber texture.

The surface morphologies of representative as-deposited films with different Ti contents are shown in Figure S2, where the evolution of the grain size with the Ti concentration is visible in the field-emission scanning electron micrographs. AZO (P0) and lightly Ti-doped TAZO-P1 exhibit relatively coalesced grains with only weakly pronounced grain boundaries, whereas the grains in heavily doped TAZO-P4 are finer and more clearly separated. This qualitative trend is consistent with the crystallite size and intensity ratio variations derived from the XRD analysis: the Scherrer evaluation shows that the (002) crystallite size decreases from about 8–9 nm for P0–P1 to smaller values at higher Ti contents, and the (002) peak-intensity ratio I_002_/I_002,as_ after FLA increases most strongly for AZO (P0), while only moderate enhancements are found for the Ti-rich films. Such microstructural changes are known to influence carrier scattering at grain boundaries;^16,24,25^ in combination with defect engineering achieved by FA and especially FLA, they help to rationalize the Ti-dependent mobility and sheet resistance behavior discussed in Section 2.2.

### 2.2 Electrical Properties

The sheet resistance data for the as-deposited and furnace- or flash-lamp-annealed samples at different parameters are presented in Figure 3. The sheet resistance of the as-deposited samples is quite high and varies between 0.06 MΩ/sq for the sample with 0.09 atom % Ti (TAZO-P1) through 0.21 MΩ/sq for the pure AZO sample to 3.05 MΩ/sq for the sample with 0.43 atom % of Ti (sample P4). It has been studied that only a small amount of Ti dopants under certain concentrations could induce more electrons and avoid acting as scattering centers.^15,16,24^ After furnace annealing, the sheet resistance is reduced to 38.24 kΩ/sq for pure AZO and increases to 371.33 kΩ/sq for the TAZO-P4 sample. The TAZO-P1 sample has a sheet resistance of about 7.66 kΩ/sq, which is five times lower compared to that of the as-deposited state. Interestingly, the lowest sheet resistance is always achieved for the TAZO-P1 samples with a small amount of Ti. The samples with the highest Ti concentrations show the highest sheet resistance. Simultaneously, these samples have the smallest average crystal size, which can partially explain the high resistivity due to carrier scattering on the grain boundaries. All samples, after FLA for 23 ms, exhibit a sharp increase in the layer conductivity in comparison with furnace annealing. The lowest sheet resistance of 754 Ω/sq is achieved for the sample annealed by FLA at the highest energy density of 70.6 J cm^–2^ and containing 0.09 at% of Ti (TAZO-P1 sample). In general, after FLA at 70.6 J cm^–2^, the sheet resistance is reduced by about one hundred times compared to the as-deposited samples and is about ten times lower than that obtained after FA. From the relation *R*_s_ ∝ 1/(*q* *n* μ *t*), where *q* is the elementary charge, *n* is the carrier concentration, μ is the mobility, and *t* is the film thickness, the observed sheet resistance trends can be understood as the combined effect of *n* and μ. For all the samples, FA and FLA lead to higher n and μ (see Table 1), reflecting improved crystallinity and reduced defect scattering. The pronounced decrease of *R*_s_ for TAZO-P1 after FLA, compared with pure AZO (P0) and heavily Ti-doped samples (P3–P4), indicates that a small Ti content optimizes the balance between donor activation and scattering: Ti^4+^ substituting Zn^2+^ contributes additional free electrons, whereas excessive Ti incorporation introduces point defects and grain-boundary disorder that strongly reduce μ.

**Figure 3:**
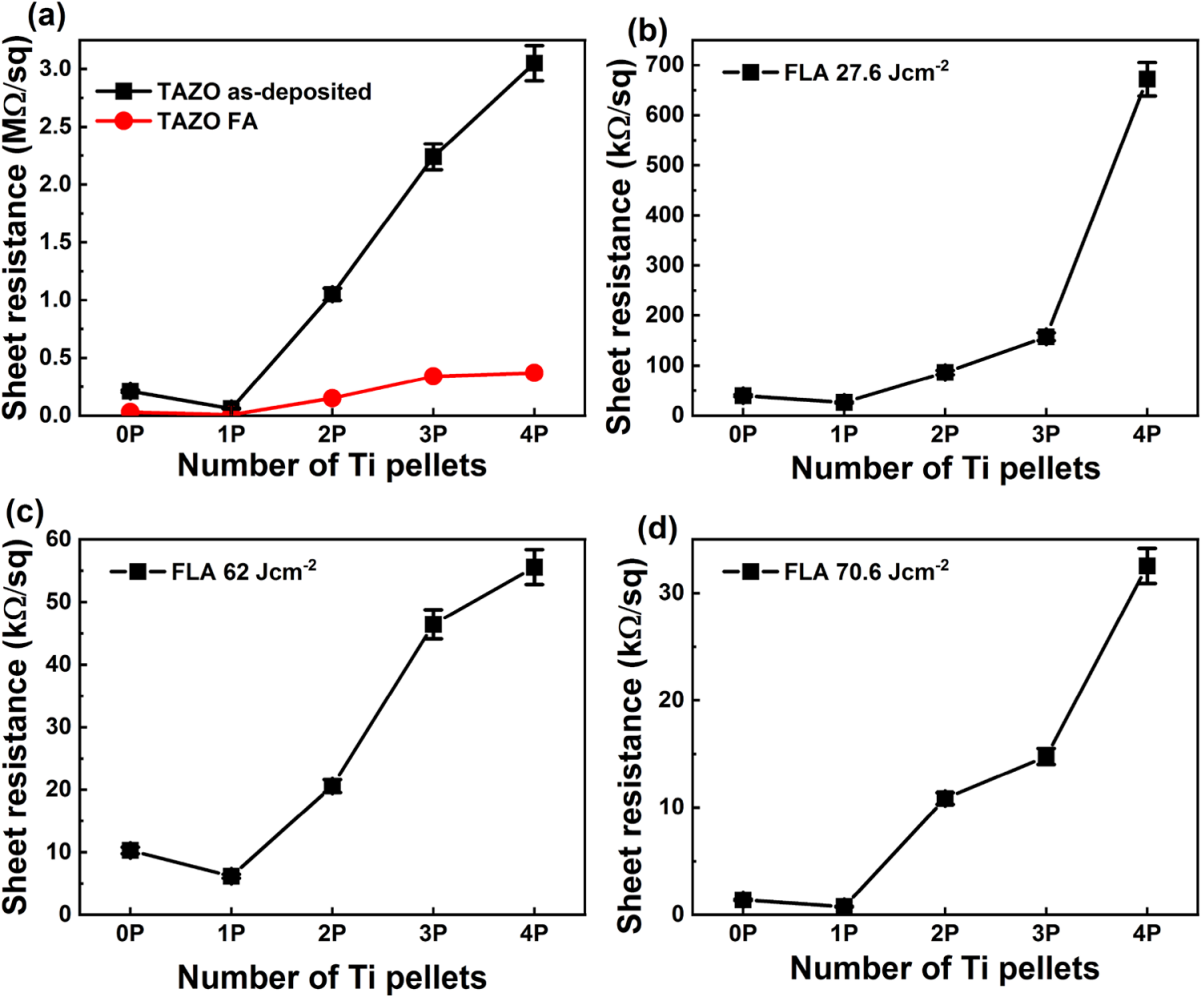
Sheet resistance as a function of the Ti concentration obtained from samples annealed by FLA for 23 ms at different energy densities is indicated in figures b-d, and (a) shows the data for the as-deposited and furnace-annealed samples.

**Table 1:** Summary of the Electrical Properties of the (T)­AZO Films before and after Annealing^a^

annealing parameters	concentration *n*(×10^18^ cm^–3^)	mobility cm^2^/(V·s)	sheet resistance	annealing parameters	concentration *n*(×10^18^cm^–3^)	mobility cm^2^/(V·s)	sheet resistance
TAZO-P0				TAZO-P3			
As-deposited	0.81	0.41	0.14 MΩ/sq	As-deposited	0.06	0.34	2.24 MΩ/sq
FA	1.31	1.03	38.24 kΩ/sq	FA	0.09	1.50	345.91 kΩ/sq
FLA 27.6 J cm^–2^	2.09	0.62	39.55 kΩ/sq	FLA 27.6 J cm^–2^	0.35	0.94	157.5 kΩ/sq
FLA 62 J cm^–2^	6.67	0.75	10.28 kΩ/sq	FLA 62 J cm^–2^	0.46	1.35	82.5 kΩ/sq
FLA 70.6 J cm^–2^	27.21	1.36	1.39 kΩ/sq	FLA 70.6 J cm^–2^	2.16	1.62	14.78 kΩ/sq
TAZO-P1				TAZO-P4			
As-deposited	1.63	0.68	0.04 MΩ/sq	As-deposited	0.06	0.25	3.05 MΩ/sq
FA	3.85	1.75	7.66 kΩ/sq	FA	0.11	1.25	371.33 kΩ/sq
FLA 27.6 J cm^–2^	2.41	0.81	26.31 kΩ/sq	FLA 27.6 J cm^–2^	0.16	0.48	672.08 kΩ/sq
FLA 62 J cm^–2^	9.57	0.86	6.19 kΩ/sq	FLA 62 J cm^–2^	0.24	0.96	219.5 kΩ/sq
FLA 70.6 J cm^–2^	38.52	1.79	0.75 kΩ/sq	FLA 70.6 J cm^–2^	0.81	1.96	32.55 kΩ/sq
TAZO-P2							
As-deposited	0.54	0.43	0.21 MΩ/sq				
FA	0.8	1.67	36.63 kΩ/sq				
FLA 27.6 J cm^–2^	0.8	1.02	59.5 kΩ/sq				
FLA 62 J cm^–2^	2.45	1.06	20.58 kΩ/sq				
FLA 70.6 J cm^–2^	3.06	1.56	10.84 kΩ/sq				

a The carrier concentration, mobility, and sheet resistance were estimated using the Hall effect measurements.

In addition, oxygen-rich growth conditions favor the formation of compensating O_i_-related acceptor defects; subsequent FA and especially FLA partially remove or reconfigure these defects, as confirmed by the PL analysis presented in Figure 5, thereby further increasing n and reducing *R*_s_. The electrical properties of the (T)­AZO films are summarized in Table 1.

We also measured the carrier concentration and carrier mobility using the Hall effect in the van der Pauw configuration. The electron concentration in the as-deposited samples is on the order of 10^17^ cm^–3^ for the TAZO-P0 and TAZO-P1 samples and on the order of 10^16^ cm^–3^ for the rest of the samples. The electron mobility is also quite low, in the range of 0.2 to 1 cm^2^/(V·s). After FA treatment in nitrogen, both the carrier concentration and carrier mobility increased in all samples. The highest electron concentration after FA is measured for the TAZO-P1 sample, and it is about 3.8 × 10^18^ cm^–3^. The most conductive samples are achieved after FLA for 23 ms. The highest electron concentration of 3.8 × 10^19^ cm^–3^ is measured for the TAZO-P1 sample with an electron mobility of about 1.79 cm^2^/(V·s). After annealing at the highest energy density, the crystallinity of the layer improved, as revealed by XRD measurements, which reduces the defect density and consequently decreases the number of scattering centers in the lattice, thereby enhancing carrier mobility. Additionally, the increased carrier concentration after FLA treatment is attributed to the reduction in the number of electrons trapped in the crystal by defects. Similar to the FA-treated sample, the sample annealed by FLA shows a continuous decrease of the carrier concentration with increasing Ti concentration. These results suggest that annealing using FLA at an optimized energy density of 70.6 J cm^–2^ (for 23 ms in N_2_ atmosphere) can not only significantly improve the carrier concentration from 3.85 × 10^18^ cm^–3^ (FA-treated) to 3.52 × 10^19^ cm^–3^ (FLA-treated) and reduce the sheet resistance from 7.66 kΩ/sq to 754 Ω/sq, but also maintain a comparable carrier mobility (FA: 1.75 cm^2^/(V·s), FLA: 1.79 cm^2^/(V·s)). This demonstrates that FLA is a highly effective and rapid postdeposition treatment, achieving superior electrical performance with a drastically reduced processing time compared to conventional furnace annealing. The underlying mechanism for this superiority, selective carrier activation with minimal structural degradation, is elucidated in the photoluminescence (PL) discussion.

### 2.3 Optical Properties

Figure 4 shows the change in the transmittance of the samples after annealing at different parameters. The transmittance spectra were measured between 300 and 800 nm, and the average value is estimated for the spectral range of 400–800 nm. The average transmittance of the as-deposited samples varies between 73.2% and 77.6%. The average transmittance of the thin films was found to decrease due to the increase in Ti content.^25^ After furnace annealing, it increased to 81.9% for the TAZO-P0 sample, and improved by 76.5% to 79.3% for the TAZO-P1 sample. For the TAZO-P1 samples, an increase in the transmittance from 76.5% for as-deposited to 79.5% is observed after FLA at 70.6 J cm^–2^ due to the increased crystallinities of the films, which is consistent with the XRD results presented in Figures 1 and 2. Also, after FLA, the highest transmittance of 81.5% is measured for the TAZO-P0 sample after flash lamp annealing at an energy density of 62 J cm^–2^. Increasing the flash energy density to 70.6 J cm^–2^ reduces the transmittance to 80.1%. The TAZO-P0 sample flashed at 70.6 J cm^–2^ also has a higher carrier concentration than the sample flashed at 62 J cm^–2^. An increase of the electron concentration can be one of the reasons for the reduced transmittance caused by light scattering on free carriers. The band gap energies of the AZO and TAZO thin films with different titanium concentrations were determined using the Tauc method by plotting (α*hv*)^2^ against the photon energy of the film (see Figure 4). The absorption coefficient (α) and energy band gap (*E*_g_) were calculated using the following Tauc’s relation

2 $$\alpha hv\approx {\left(\mathrm{hv}-E_{g}\right)}^{2}$$

where *hv* is the photon energy, and *E*_g_ can be obtained by extrapolating the linear line of the curves toward the energy *x*-axis. In most cases, FLA causes an increase of the optical band gap due to defect reduction and activation of Al donors.^16,26,27^ The influence of Ti codoping on the band gap change after annealing cannot be clearly determined. Most probably, the Ti concentration is too low to significantly change the band gap of the fabricated TAZO films. The changes in the transmission and band gap are summarized in Table 2.

**Figure 4:**
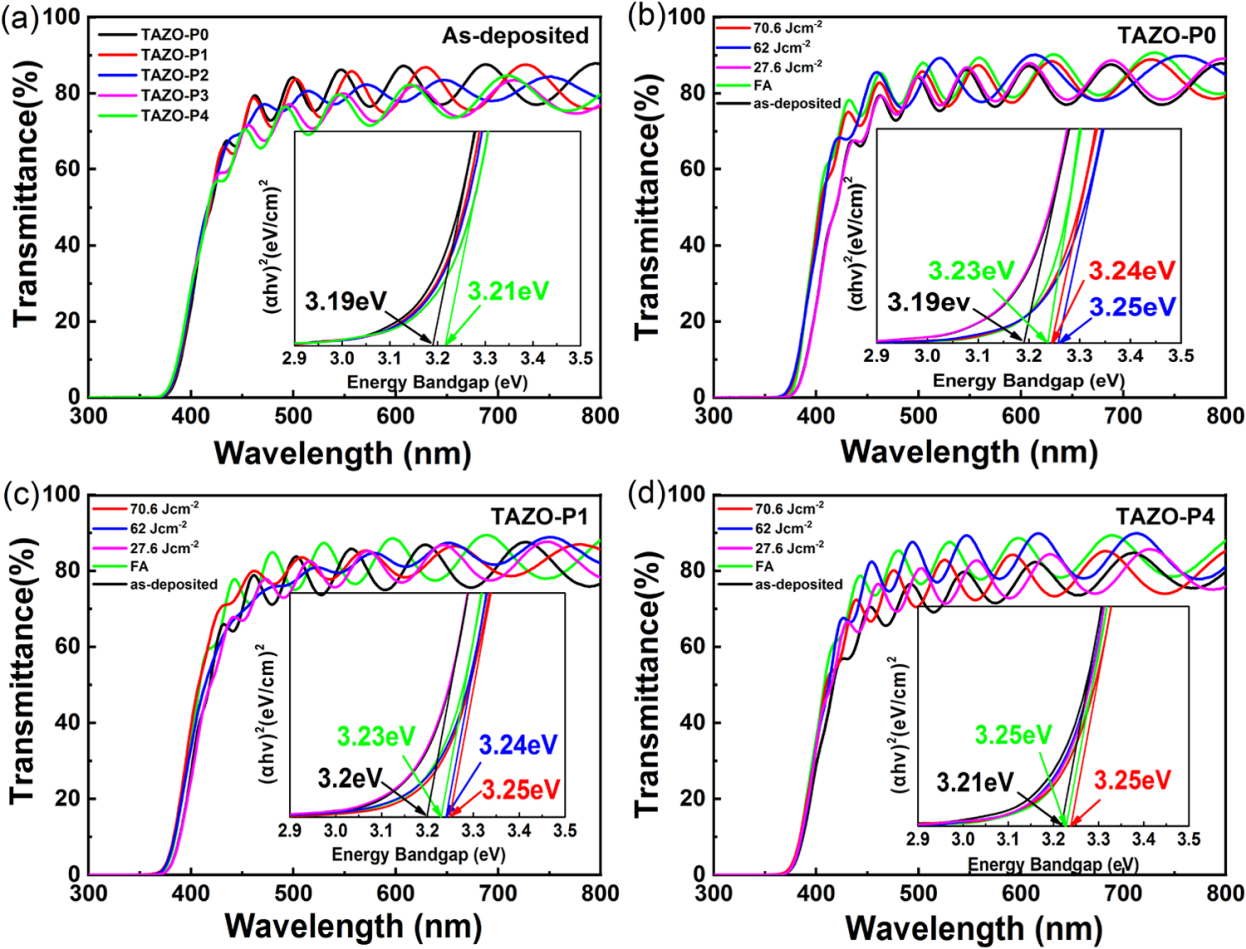
Transmission of the (T)­AZO films on the glass substrate, and the estimated band gap changes for the samples annealed at different conditions (see the insets) are indicated in figures (a–d). The band gap was estimated by fitting the experimental results to a Tauc plot.

**Table 2:** Transmission and Band Gap of the (T)­AZO Films in the As-Deposited Stage and after Annealing in N_2_ Flow^a^

annealing parameters	average transmittance (%)	band gap (eV)	annealing parameters	average transmittance (%)	band gap (eV)
TAZO-P0			TAZO-P3		
As-deposited	77.6	3.19	As-deposited	73.4	3.21
FA	81.9	3.23	FA	81.8	3.23
FLA 27.6 J cm^–2^	78.5	3.19	FLA 27.6 J cm^–2^	75.3	3.22
FLA 62 J cm^–2^	81.5	3.25	FLA 62 J cm^–2^	74.7	3.23
FLA 70.6 J cm^–2^	80.1	3.24	FLA 70.6 J cm^–2^	79.3	3.22
TAZO-P1			TAZO-P4		
As-deposited	76.6	3.2	As-deposited	73.2	3.21
FA	79.3	3.23	FA	80	3.22
FLA 27.6 J cm^–2^	77	3.2	FLA 27.6 J cm^–2^	75.1	3.22
FLA 62 J cm^–2^	79	3.24	FLA 62 J cm^–2^	79.8	3.22
FLA 70.6 J cm^–2^	79.5	3.25	FLA 70.6 J cm^–2^	75.4	3.24
TAZO-P2					
As-deposited	76.5	3.2			
FA	82.4	3.21			
FLA 27.6 J cm^–2^	77.6	3.21			
FLA 62 J cm^–2^	75.8	3.23			
FLA 70.6 J cm^–2^	79.6	3.23			

a The band gap values were obtained by Tauc plot fitting of the experimental data.

## 3 Discussion

In order to explain the variation in the electrical properties of (T)­AZO films with changing Ti concentration, we analyzed both the process parameters and sample composition. Based on the EDS results, the concentration of Al in the layer is about 1.5 atom %, which is lower than the nominal sputter target composition with about 2 atom % of Al. According to the literature, AZO films fabricated with optimum deposition and postdeposition annealing parameters may have a sheet resistivity in the range of 20 Ω/sq,^28^ and the lowest resistivity of about 2 × 10^–4^ Ω·cm is achieved for FLA-treated AZO films.^29^ The titanium-doped AZO with Ti and Al concentrations of 0.59 atom % and 3.43 atom %, respectively, may have a resistivity as low as 9 × 10^–4^ Ω·cm.^16^ In our case, the electrical properties of both AZO (TAZO-P0) and TAZO films are indeed worse than these state-of-the-art values. Our lowest resistivity is 9.05 × 10^–2^ Ω·cm for TAZO-P1 after FLA, which is 1–2 orders of magnitude higher than the best values reported for optimized AZO thin films grown by RF sputtering and self-bias control.^40^ This difference is expected because our layers were deliberately grown by DC sputtering at 4.0 × 10^–2^ Torr in an oxygen-rich Ar/O_2_ plasma, which yields relatively small grains and a high density of compensating oxygen-related defects. Within this intentionally nonoptimized growth window, the aim of the present work is therefore not to achieve record-low resistivity but to evaluate how ms-range FLA compares with FA in improving the microstructure and optoelectronic properties of ZnO-based TCOs. Applying this strongly nonequilibrium FLA process, we are able to reduce the sheet resistance by about 2 orders of magnitude while slightly enhancing the optical transmittance of the films. A closer look at the composition of the films obtained from the EDS analysis in Table S3 reveals that the oxygen content in the as-deposited TAZO-P0 sample is on the order of 59 atom %, while in the TAZO-P1 to TAZO-P4 samples it is about 55 atom %. The high oxygen content suggests that the deposition process occurred in oxygen-rich conditions. It is known that a high concentration of oxygen vacancies (double donors) and Zn interstitials improves the electrical conductivity of ZnO-based layers.^13,30^ In contrast, high oxygen content increases the concentration of oxygen interstitials and reduces the electrical conductivity.^32,36^ Our samples were annealed in continuous N_2_ flow, which may slightly reduce the concentration of oxygen in the sample. Nevertheless, due to the Zn/O ratio being smaller than 1, all Zn interstitials can be fully oxidized, and free oxygen can remain in the sample at the interstitial position, reducing the average electron concentration. The formation of oxygen interstitials during annealing is well visible in the PL spectra presented in Figure 5.

**Figure 5:**
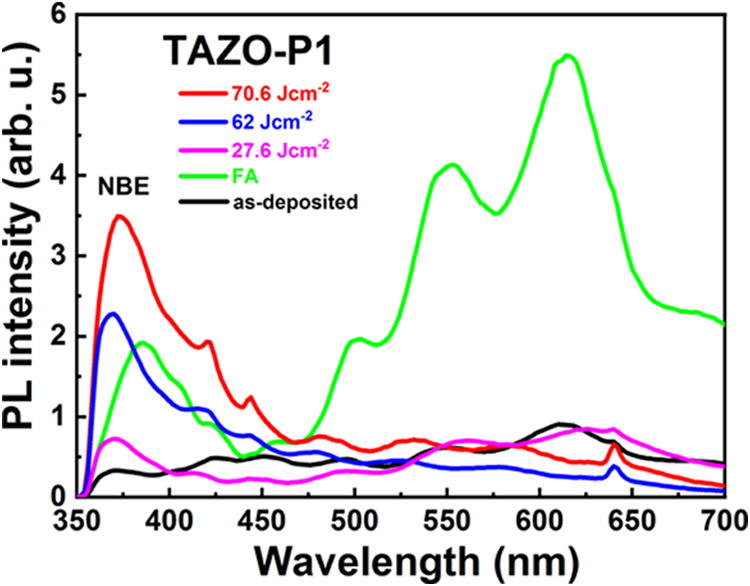
PL spectra recorded from the TAZO-P1 sample after the FA and FLA processes. The samples were excited with a 320 nm laser at room temperature. The multipeak structure of the PL emission is due to interference within the coating.

The PL spectrum of the TAZO-P1 sample annealed with FA is dominated by red emission at about 600 nm. The red emission above 600 nm is typically assigned to oxygen interstitials, i.e., radiative transition between the conduction band or shallow donors and oxygen interstitial-related acceptor level.^32^ The PL spectrum of the sample with the highest electron concentration (TAZO-P1 after FLA at 70.6 J cm^–2^) is dominated by near-band gap emission. However, the as-deposited sample exhibited almost no PL emission. The obtained PL results are consistent with the structural and electrical data. The highest crystallinity is achieved after the FLA process at the highest allowed energy density.

A comparison between FA and FLA reveals distinct differences in the electrical, optical, and photoluminescence (PL) properties, which can be closely linked to the defect states modulated by each annealing method. The peak deconvolution of the PL spectra of the TAZO-P1 sample annealed by FLA or FA is presented in Figure S3a,b, respectively. Exact fitting is difficult due to interferences within the film. In the case of the FLA-treated sample, the UV-blue emission is asymmetric and can be fitted with two Gaussian functions located at about 373 and 404 nm. The UV peak corresponds to the near-band gap emission, while the peak at about 404 nm can be commonly ascribed to the radiative recombination of electrons between the neutral zinc interstitial and the valence band. In addition, samples annealed with FLA exhibit weak green emission at about 506 nm, which can be due to zinc vacancies, oxygen vacancies, or defect complexes. Since the signal is quite weak, we can assume a low concentration of such defects. After FA, the NBE is recorded at about 380 nm, and the Zn_i_-related peak is at about 400 nm. Among these two peaks, the red emission near 600 nm, typically attributed to O_i_, dominates the PL spectrum of the FA-treated sample.^32–34^ Oxygen interstitials are known to act as deep acceptor defects that trap free carriers, thereby significantly degrading the n-type conductivity.^13,34^ In contrast, Zn_i_ and V_O_ often behave as shallow donors and may contribute positively to the carrier concentration if they are properly controlled.^33,34^

The wave-like modulations near 600 nm in the PL spectrum of the FA-treated sample may also originate from optical interference effects, which become more pronounced in relatively thick films. In the present case, the film thickness is approximately 1.2 μm, as determined using an α-step profilometer.^37^ Collectively, these results indicate that FLA suppresses O_i_-related defect formation even for samples grown at oxygen-rich conditions,^13,34^ while activating donor-like defects (V_O_, Zn_i_),^32,33^ thereby enabling a substantial increase in carrier concentration without excessive structural degradation. Both optical, electrical, and structural data confirm that the millisecond-scale FLA allows defect engineering in ZnO-based TCO and significantly outperforms long-duration FA.^28,31^ Unlike FA, the short, high-energy FLA pulse prevents excess oxygen incorporation and promotes rapid defect reconfiguration, resulting in enhanced crystallinity and optoelectronic performance.^20,28,31^ This trend is consistent with earlier ITO studies,^38^ where even submillisecond FLA (0.5 ms) yielded electrical properties comparable to furnace annealing for 1 h at 200–300 °C. In our case, the 23 ms pulse provided a sufficient thermal budget to selectively activate donor-like defects (e.g., Zn_i_) while suppressing deep acceptor O_i_ formation, as confirmed by PL analysis. In contrast, prolonged furnace annealing promotes a higher density of O_i_ and degrades the electrical conductivity of the sample. In terms of the economic aspect of different annealing methods, FLA is a few times cheaper than conventional annealing. According to the cost calculations performed for the glass industry, the average costs of the FLA treatment of 1 m^2^ of glass are in the range of 0.12 to 0.36 Euro/m^2^, while the rapid thermal processing providing similar product performance as FLA-treated samples is on the order of 0.70 Euro/m^2^ (longer annealing requires higher energy consumption and higher costs).^39^ The calculation was made for the glass industry for the activation of low-emissivity coating. Hence, FLA provides the possibility for efficient defect engineering and dopant activation and is a cost-effective method.

## 4 Conclusions

In this work, (T)­AZO thin films were deposited on glass substrates by reactive DC magnetron sputtering under oxygen-rich conditions and subsequently annealed either by conventional furnace annealing (FA) or millisecond-range flash lamp annealing (FLA). FLA significantly enhanced the crystallinity, optical transmittance, and electrical conductivity of films. The TAZO film with a Ti concentration of 0.09 atom % exhibited the highest mobility and lowest resistivity (9.05 × 10^–2^ Ω·cm) after FLA for just 23 ms at an energy density of 70.6 J cm^–2^, together with an average transmittance of about 80%. Although these values are higher than the best resistivities reported for optimized AZO, the key result is that FLA reduces the sheet resistance by approximately 2 orders of magnitude relative to the as-deposited state and by roughly 1 order of magnitude compared to FA, while consuming about three times less energy than conventional heating. Although the current study primarily focused on the influence of different thermal treatments on the optical and electrical properties of (T)­AZO films, the direct influence of Ti codoping on the electrical properties of TAZO films remains not fully understood. This aspect warrants further investigation, and one solution would be to apply Zn-rich sputtering conditions by varying the oxygen content in the plasma or using different Al concentrations. In general, the electrical conductivity of flashed films is several orders of magnitude higher than that of films treated by conventional furnace annealing.

Overall, our results demonstrate that a millisecond-range FLA is an efficient, cost-effective nonequilibrium process for defect engineering and dopant activation in ZnO-based transparent conducting oxides grown under technologically relevant oxygen-rich DC sputtering conditions.

## 5 Methods and Characterization

### 5.1 DC Sputtering Method

Al-doped ZnO (AZO) and Ti–Al-codoped ZnO (TAZO) thin films on glass substrates were deposited by a reactive DC magnetron sputtering technique using a 99.9% AZO target (2 wt % Al_2_O_3_, 98 wt % ZnO, 2-in. diameter and 0.125-in. thickness) on which 99.9% Ti pellets (Krtlab, 99.9% purity, diameter 3 mm, 3 mm thick) were mounted in the erosion area of the target surface to achieve different Ti doping concentrations (see Figure 6b). The Ti doping concentrations were controlled by changing the number of Ti pellets from zero to four. 15 mm × 15 mm glass substrates with a thickness of 0.5 mm were placed at a distance of 7 cm from the target. Prior to AZO film deposition, the substrates were ultrasonically cleaned in acetone, ethyl alcohol, and deionized water for 5 min each and then dried with a purge of high-purity N_2_ gas. The base pressure in the deposition chamber was 5.0 × 10^–6^ Torr, and sputtering was conducted at a working pressure of 4.0 × 10^–2^ Torr. In our system, this pressure ensures a stable discharge even when several Ti pellets are mounted on the AZO target, which is essential for obtaining reproducible films across the full Ti concentration series (P0–P4). The glass substrates were placed on a rotating disk and heated to 200 °C prior to the sputtering process. In order to clean the sputtering target from any contamination, presputtering was carried out for 10 min at 50 W in a 25 sccm Ar gas flow. The deposition time of the AZO and TAZO films was fixed at 30 min, and the flow rates of Ar (purity 99.99%) and O_2_ were 25 and 5 sccm, respectively. The sputtering plasma power was 200 W, and the layer thickness of the as-obtained thin films was roughly 1.2 μm. More details about the sputtering process can be found in ref 21. These DC sputtering conditions (4.0 × 10^–2^ Torr, Ar/O_2_ = 25/5 sccm) are intentionally oxygen-rich and at a relatively high working pressure; although they do not yield the lowest possible resistivity for AZO, they are representative of large-area industrial deposition and provide a convenient starting point with high defect sensitivity to evaluate the impact of FA and FLA on (T)­AZO films.

**Figure 6:**
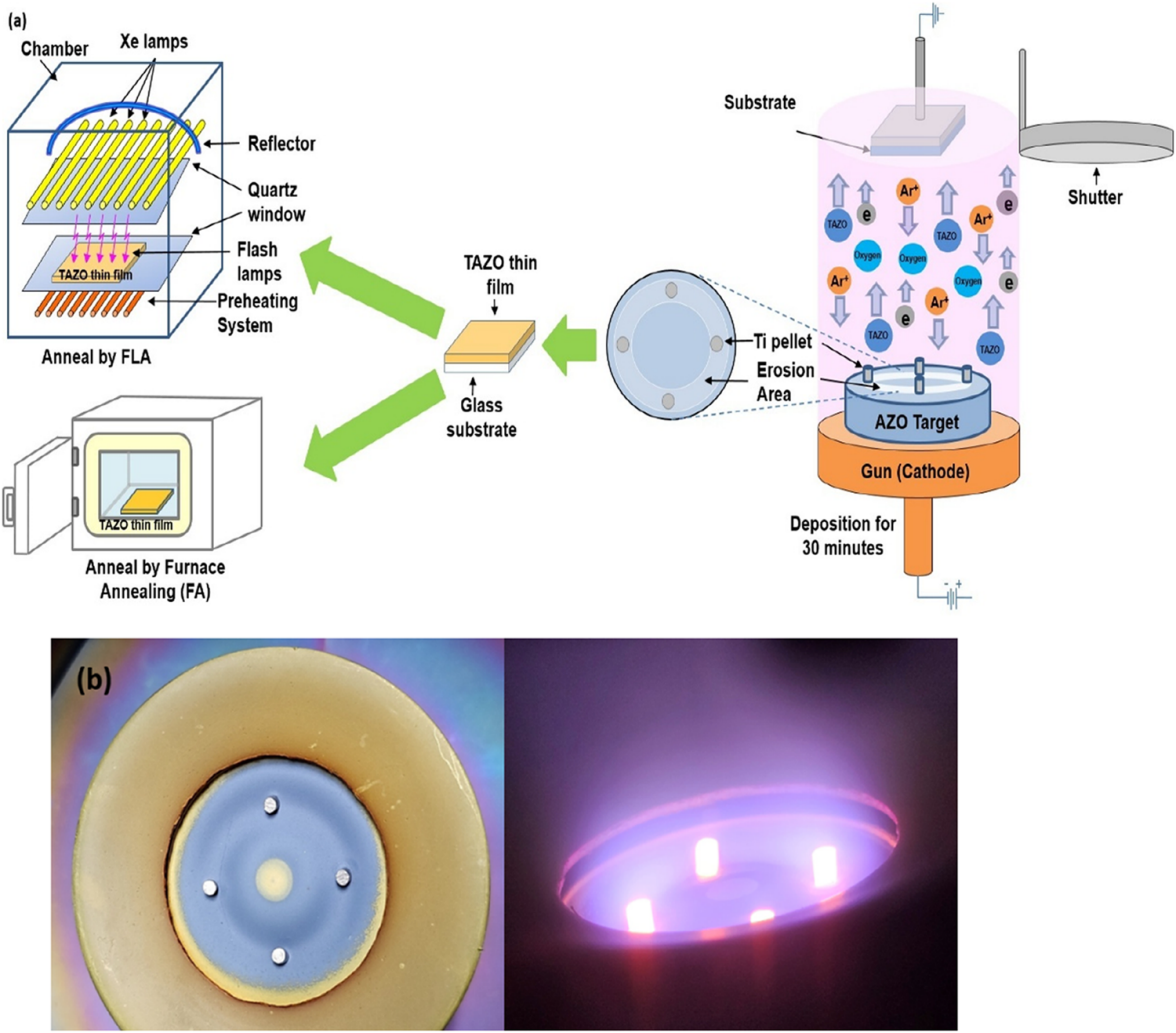
(a) Schematic diagram of the deposition of Ti–Al-codoped ZnO (TAZO) film by DC magnetron sputtering with Ti pellets on an AZO target, and postannealing by flash lamp annealing (FLA) or furnace annealing (FA). (b) Photographs of the sputtering target with Ti pellets mounted in the erosion area (left) and the plasma generated during the DC sputtering process (right).

### 5.2 Postannealing: Flash Lamp Annealing (FLA) and Furnace Annealing

Postdeposition annealing was performed using either a high-intensity Xenon flash lamp system at Blitz-Lab HZDR or conventional furnace annealing. The annealing was performed in a continuous flow of N_2_ gas. The flash energy densities were 27.6, 62, and 70.6 J cm^–2^ with a flash pulse length of 23 ms. Annealing at a lower energy density has no influence on the properties of the coating, while annealing at a higher energy density causes delamination of the layer from the glass substrate. During the FLA process, most of the energy is deposited in the coating, keeping the glass substrate at a much lower temperature. Therefore, FLA can be successfully applied to anneal thin films coated on temperature-sensitive substrates, like polymers or glass. Conventional furnace annealing (FA) was carried out for 30 min at 500 °C.

### 5.3 Characterization

The structural properties of the as-grown and annealed samples were investigated using a D8 Advance X-ray diffractometer (XRD) (Bruker, Karlsruhe, Germany), with a Cu-Kα source and a scintillation counter. Bragg–Brentano geometry was applied for diffraction angles ranging from 20° to 80°, with a scan rate of 2°/min.

The elemental compositions of the samples were analyzed using energy-dispersive X-ray spectroscopy (EDS) equipped with a field-emission scanning electron microscope (FE-SEM, Carl Zeiss SIGMA 300). Representative EDS spectra and quantified compositions for TAZO-P0–P4 are provided in Table S3.

In order to determine the optical band gap of the coating, photoluminescence (PL) spectroscopy and transmission measurements were performed. PL was measured using a 320 nm diode-pumped solid-state laser with a power of 20 mW and a 340 nm longpass edge filter for excitation. The PL signal was recorded using a 550 iHR Horiba spectrometer and a Hamamatsu H7732-10 photomultiplier. In order to increase the signal-to-noise ratio, a lock-in amplifier was used, and the laser light was chopped at a frequency of 25 Hz. PL measurements were performed at room temperature. The transmission spectra were obtained in the spectral range of 300 to 800 nm using a SolidSpec-3700i UV–VIS-NIR spectrometer (Shimadzu). The band gap of the coating was determined using Tauc plot fitting.

The electrical properties (carrier concentration and carrier mobility) of the thin films were determined using Hall effect measurements in the van der Pauw configuration at room temperature. These Hall measurements provided the carrier concentration and mobility values used to determine the resistivity and sheet resistance, as summarized in Figure 3 and Table 1.

A schematic diagram of the thin-film growth and FLA of the (T)­AZO on a glass substrate is shown in Figure 6a.

## Supplementary Material



## Notes

EDX data on the composition of the layer, and a detailed analysis of XRD data (PDF)

## Abbreviations

AZO: Al-doped ZnO
EDS: energy-dispersive X-ray spectroscopy
*E*_g_: band gap energy
FA: furnace annealing
FE-SEM: field-emission scanning electron microscope
FLA: flash lamp annealing
ITO: indium tin oxide
NBE: near-band-edge emission
O_i_: oxygen interstitial
PL: photoluminescence
Rs: sheet resistance
TCO: transparent conductive oxide
TAZO: Ti–Al-codoped ZnO
UV–Vis: ultraviolet–visible
V_O_: oxygen vacancy
V_Zn_: zinc vacancy
Zn: zinc interstitial
