# Supplementary material for: Effect of Flash Lamp and Furnace Annealing on the Electrical and Optical Properties of Ti–Al-Codoped ZnO Films Deposited by DC Magnetron Sputtering
Source: ACS Omega. 2026 May 26;11(22):32060–70. doi: 10.1021/acsomega.5c11669 (PMC13261442; doi:10.1021/acsomega.5c11669)
Supplement: Supplementary file 1 [file ao5c11669_si_001.pdf]

Supplementary Information

Effect of flash lamp and furnace annealing on electrical and optical properties of Ti-Al co-doped ZnO films deposited by DC magnetron sputtering

Mingyu Kim[1,2,3], Guoxiu Zhang[3,4], Yoon-Young Huh[1], Chang-Hyeon Jo[1], Fabian Ganss[3], Mohsin Saleem[5], Shengqiang Zhou[3], Jung-Hyuk Koh[1,2], and Slawomir Prucnal[3,*]

[1]Department of Intelligent Energy and Industry, Chung-Ang University, Heukseok-ro, Dongjak-gu, Seoul 06974, Republic of Korea

[2]School of Electrical and Electronic Engineering, Chung-Ang University, Seoul 06974, Republic of Korea

[3]Helmholtz-Zentrum Dresden-Rossendorf, Institute of Ion Beam Physics and Materials Research, Bautzner Landstraße 400, 01328 Dresden, Germany

[4]Dresden University of Technology, 01062 Dresden, Germany

[5]School of Chemical and Materials Engineering (SCME), National University of Sciences & Technology (NUST), H-12, Islamabad 44000, Pakistan

*Corresponding authors: s.prucnal@hzdr.de (S. Prucnal)

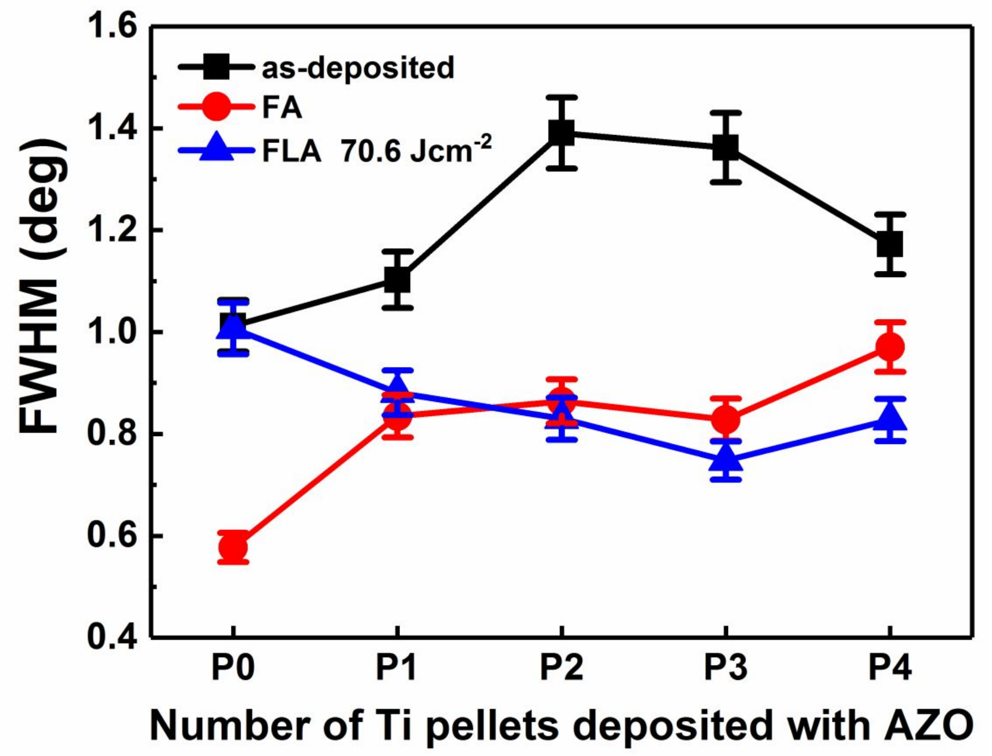

Figure S1. Full width at half maximum (FWHM) of the (002) diffraction peak as a function of Ti-concentration obtained from as-deposited sample and samples after furnace annealing (FA) and flash lamp annealing (FLA).

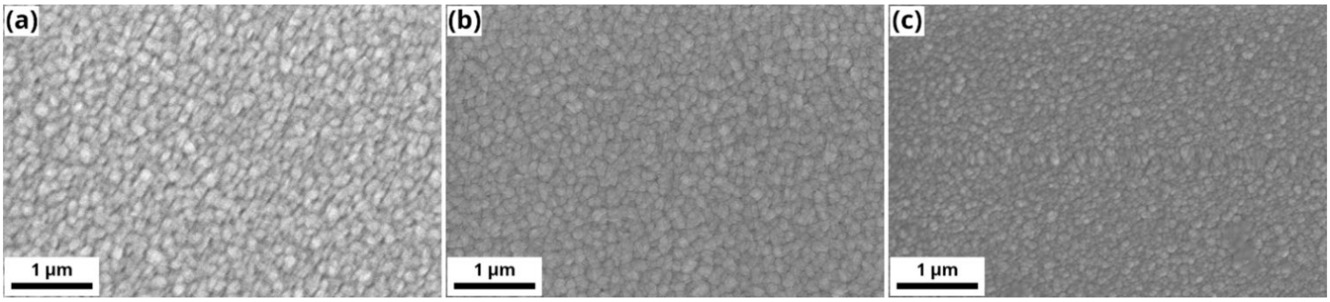

Figure S2. FESEM images of as-deposited (T)AZO thin films with varying Ti concentrations: (a) AZO (P0), (b) TAZO-P1, (c) TAZO-P4.

Supplementary Information

The surface morphology indicates that AZO (P0) and lightly Ti-doped TAZO-P1 films consist of relatively large, closely packed grains, whereas the more heavily Ti-doped TAZO-P4 sample exhibits smaller, more clearly separated grains. This qualitative difference between lightly and heavily Ti-doped films is broadly consistent with the Scherrer-derived crystallite sizes from XRD (Figure 2), which decrease upon Ti incorporation compared with AZO and lightly doped TAZO-P1.

Table S1. The EDS (Energy-Dispersive X-ray Spectroscopy) based atomic composition of the as-deposited AZO and TAZO thin films.

Map Sum Spectrum — **TAZO-P0**

Element	Signal Type	Line	Apparent Concentration	k Ratio	Wt%	Wt% Sigma	Atomic %	Standard Name	Factory Standard	Standardization Date
O	EDS	K series	60.16	0.20244	26.96	0.19	59.61	SiO2	Yes	
Al	EDS	K series	1.38	0.00990	1.13	0.07	1.49	Al2O3	Yes	
Zn	EDS	L series	100.27	1.00273	71.91	0.20	38.91	Zn	Yes	
Total					100.00		100.00			

Map Sum Spectrum — **TAZO-P1**

Element	Signal Type	Line	Apparent Concentration	k Ratio	Wt%	Wt% Sigma	Atomic %	Standard Name	Factory Standard	Standardization Date
O	EDS	K series	47.21	0.15888	23.62	0.18	55.31	SiO2	Yes	
Al	EDS	K series	1.21	0.00871	1.10	0.06	1.53	Al2O3	Yes	
Ti	EDS	K series	0.21	0.00214	0.12	0.05	0.09	Ti	Yes	
Zn	EDS	L series	99.45	0.99454	75.16	0.19	43.07	Zn	Yes	
Total					100.00		100.00			

Map Sum Spectrum — **TAZO-P4**

Element	Signal Type	Line	Apparent Concentration	k Ratio	Wt%	Wt% Sigma	Atomic %	Standard Name	Factory Standard	Standardization Date
O	EDS	K series	49.24	0.16570	23.80	0.12	55.48	SiO2	Yes	
Al	EDS	K series	1.32	0.00950	1.14	0.04	1.58	Al2O3	Yes	
Ti	EDS	K series	1.08	0.01078	0.56	0.04	0.43	Ti	Yes	
Zn	EDS	L series	102.57	1.02574	74.50	0.12	42.50	Zn	Yes	
Total					100.00		100.00			

Supplementary Information

Table S1 shows the composition of AZO and TAZO films at different titanium concentrations controlled by the number of pellets. We measured atomic percentages of Al, Zn, and O in AZO (TAZO-P0) of 1.5%, 38.9%, and 59.6%, respectively. The atomic percentage of titanium was measured to be about 0.1% for TAZO-P1 and about 0.4% for TAZO-P4. We can thus assume that the titanium concentration is roughly proportional to the number of titanium pellets placed on the AZO sputter target.

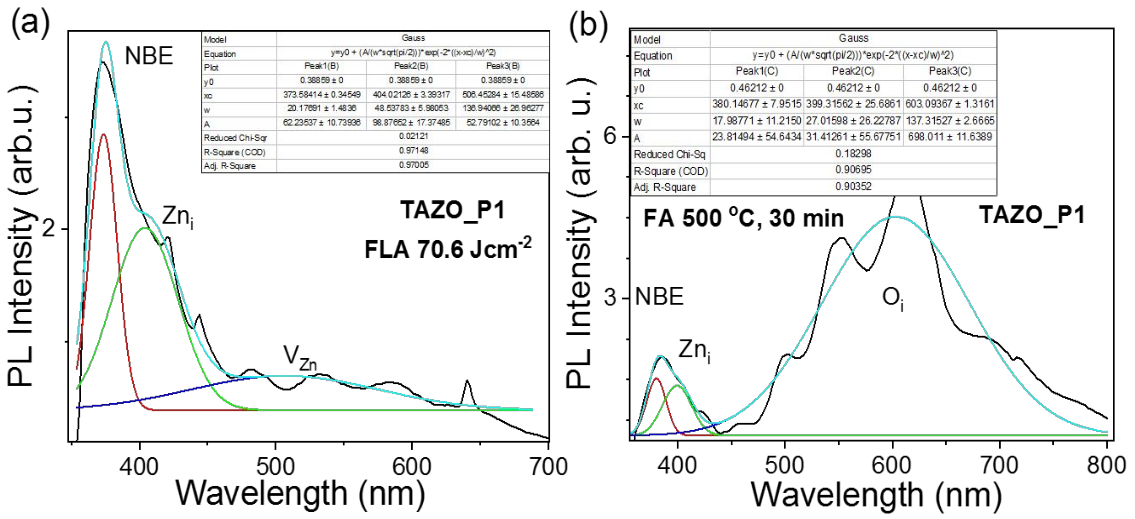

Figure S3. Fitting of the PL spectra obtained from TAZO-P1 samples after FLA (a) and after FA (b). The fitting was performed using three Gaussian functions.

Table S2. Integrated area I_{002} of the ZnO (002) diffraction peak for (T)AZO films with different Ti contents and annealing conditions.

Sample	As-deposited	FLA 27.6 J cm^{-2}	FLA 62.0 J cm^{-2}	FLA 70.6 J cm^{-2}	FA 500 °C
P0	70.53	90.82	105.83	227.49	88.63
P1	178.96	171.1	201.22	280.02	265.19

P2	210.88	133.13	288.21	217.34	245.53
P3	186.85	160.95	161.46	175.8	173.98
P4	135.8	169.83	241.76	227.32	230.11

Table S3 Normalized area $I_{002}/ I_{002,as}$ of the ZnO (002) peak, highlighting the enhancement factors after FLA and FA.

**Sample**	**As-deposited**	**FLA 27.6 J cm⁻²**	**FLA 62.0 J cm⁻²**	**FLA 70.6 J cm⁻²**	**FA 500 °C**
P0	1.00	1.28	1.5	3.22	1.25
P1	1.00	0.95	1.12	1.56	1.48
P2	1.00	0.63	1.36	1.03	1.16
P3	1.00	0.86	0.86	0.94	0.93
P4	1.00	1.25	1.78	1.67	1.69
